# Investigation of Vitamin D_2_ and Vitamin D_3_ Hydroxylation by *Kutzneria albida*

**DOI:** 10.1002/cbic.202100027

**Published:** 2021-05-04

**Authors:** Lisa Marie Schmitz, Alina Kinner, Kirsten Althoff, Katrin Rosenthal, Stephan Lütz

**Affiliations:** ^1^ Chair for Bioprocess Engineering Department of Biochemical and Chemical Engineering TU Dortmund University Emil-Figge-Straße 66 44227 Dortmund Germany

**Keywords:** biotransformations, cyclodextrins, hydroxylation, *Kutzneria albida*, vitamin D

## Abstract

The active vitamin D metabolites 25‐OH−D and 1α,25‐(OH)_2_−D play an essential role in controlling several cellular processes in the human body and are potentially effective in the treatment of several diseases, such as autoimmune diseases, cardiovascular diseases and cancer. The microbial synthesis of vitamin D_2_ (VD_2_) and vitamin D_3_ (VD_3_) metabolites has emerged as a suitable alternative to established complex chemical syntheses. In this study, a novel strain, *Kutzneria albida*, with the ability to form 25‐OH−D_2_ and 25‐OH−D_3_ was identified. To further improve the conversion of the poorly soluble substrates, several solubilizers were tested. 100‐fold higher product concentrations of 25‐OH−D_3_ and tenfold higher concentrations of 25‐OH−D_2_ after addition of 5 % (*w*/*v*) 2‐hydroxypropyl β‐cyclodextrin (2‐HPβCD) were reached. Besides the single‐hydroxylation products, the human double‐hydroxylation products 1,25‐(OH)_2_−D_2_ and 1,25‐(OH)_2_−D_3_ and various other potential single‐ and double‐hydroxylation products were detected. Thus, *K. albida* represents a promising strain for the biotechnological production of VD_2_ and VD_3_ metabolites.

## Graphical abstract

**The microbial transformation** of vitamin D to the active forms by a two‐step hydroxylation represents a promising alternative to complex chemical syntheses. The ascomycete *K. albida* was shown to catalyze vitamin D hydroxylation by forming diverse single‐ and double‐hydroxylation products. The efficiency of the reaction could be drastically increased by the addition of cyclodextrin derivatives to the biotransformation.

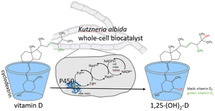

## Introduction

**Scheme 1:**
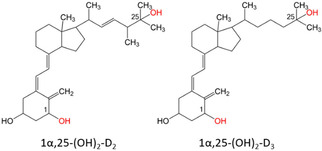
Structure of the human VD_3_ and VD_2_ metabolites 1,25‐(OH)_2_−D_2_ and 1,25‐(OH)_2_−D_3_. Hydroxylation is first introduced in the C25 position in the liver followed by a hydroxylation in C1 position in the kidney.^[11]^

Cytochrome P450 monooxygenases (P450s) constitute a large family of heme *b*‐containing enzymes that are present in nearly all organisms from all domains of life. By activating dioxygen molecules, they catalyze the monooxygenation of diverse substrates. Due to their high catalytic diversity and regioselectivity, they open a novel chemical space and pose an efficient alternative to chemical synthesis for the production of many functional molecules, such as fine chemicals or pharmaceuticals. In humans, P450s are involved in diverse biological processes, such as the degradation of xenobiotics or the synthesis and activation of hormones,^[1]^ for example, steroids or vitamin D_3_ (VD_3_). Vitamin D forms a group of fat‐soluble, inactive prohormones that can either be synthesized naturally in the human body from 7‐Dehydrocholesterol (7‐DHC) by ultraviolet radiation (cholecalciferol/VD_3_) or can be acquired by dietary supplementation (ergocalciferol/VD_2_ and cholecalciferol/VD_3_).^[2]^ Both forms, VD_2_ and VD_3_, show biological activity in the human body with VD_3_ having a higher potency compared to VD_2_.^[3]^ Vitamin D has a significant role in the control of several cellular processes, such as the calcium and phosphate homeostasis,^[4]^ regulation of gene transcription^[5]^ and the regulation of cell proliferation and differentiation.^[6]^ Vitamin D is necessary for the maintenance of bone health,^[7]^ and has moreover been linked to reduce the risk of several autoimmune diseases,^[8]^ cardiovascular diseases,^[9]^ and the inhibition of cancer cell proliferation.^[10]^ To induce the desired effect in metabolism, VD_3_ and VD_2_ have to be transformed into their active forms by the insertion of two hydroxy groups. Insertion of a first hydroxylation at the C25‐position results in 25‐OH−D and a second hydroxylation at the C1 position in 1α,25‐(OH)_2_−D (Scheme 1).

It is known, that CYP2R1 and CYP27A1 convert vitamin D into 25‐OH−D in the liver and CYP27B1 in the kidney further hydroxylates 25‐OH−D to form 1α,25‐(OH)_2_−D.^[11]^ To counteract vitamin D deficiency of a large target population, the food supplementation with VD_2_ or VD_3_ becomes more important.^[12]^ The vitamin D therapy market already reached about US$ 1.9 billion by 2019 and is expected to reach about US$ 3.3 billion by 2024 with an annual growth rate of 11 %.^[13]^ As the supplementation with VD_2_ or VD_3_ does not provide the active forms for patients suffering from severe liver or kidney disease and subsequent enzyme deficiencies, the production of the hydroxylated metabolites is of relevance. Compared to the traditionally used complex chemical synthesis, which requires many expensive reaction steps,^[14]^ the microbial biotransformation to 25‐OH−D and 1α,25‐(OH)_2_−D offers a promising alternative. Several bacterial wild‐type strains and P450 heterologous expression strains are described catalyzing both C25 and C1 hydroxylation of VD_2_, VD_3_ ^[15]^ and of precursor molecules such as the Grundmann's ketone.^[16]^ The highest 25‐OH−D_3_ production rate described to date was achieved by a recently published whole‐cell biotransformation with *Bacillus cereus* zju 4–2, with 25.9 mg L^−1^ h^−1^.^[15d]^ Low product titers and space‐time yields (STY), however, make the microbial conversion still not feasible.

An overall hurdle in performing biotransformations with P450s is the typically high hydrophobicity of substrates and therefore low solubility in the aqueous phase. This often leads to a limited availability of substrate to the biocatalyst and reduced cellular uptake, thus hindering the overall biotransformation process. Common strategies to overcome this hurdle and to increase substrate availability are the use of surface active agents in form of emulsifiers,^[17]^ cyclic oligosaccharides,^[18]^ or biphasic systems.^[19]^ Drawbacks of especially emulsifiers and organic solvents are their negative impact on cell integrity and viability. The tolerance of the cells towards cyclic oligosaccharides, also known as cyclodextrins, is often higher. Cyclodextrins possess a hydrophilic outside surface and a large hydrophobic internal cavity which can encapsulate the hydrophobic molecule.^[20]^ Due to this complex formation, they act as carrier molecules and therefore can drastically improve solubility and the solubilization kinetics of hydrophobic molecules.^[21]^ Several examples are known, in which the usage of cyclodextrins improved the microbial bioconversion of VD_2_ and VD_3_.[^15d^, ^22^]

In this study, we identified the microorganism *Kutzneria albida*, to be able to hydroxylate both VD_2_ and VD_3_ in the C25 and in the C1 position. As variation of the medium did not significantly increase the product formation, different solubilizers were tested to increase the solubility of VD_2_ and VD_3_. It was suggested that the poor solubility lowers the availability of the substrates to the biocatalyst and thus results in an inefficient conversion. The addition of the solubilizers methyl β‐cyclodextrin (MβCD), 2‐hydroxypropyl β‐cyclodextrin (2‐HPβCD), and 2‐hydroxypropyl γ‐cyclodextrin (2‐HPγCD) significantly increased the biotransformation of both VD_2_ and VD_3_. Especially, high yields of 25‐OH−D_3_ were obtained. These results make *K. albida* a new potential strain for the microbial production of VD_2_ and VD_3_ metabolites.

## Results

### Identification of a novel strain for the bioconversion of VD_2_ and VD_3_

Recently, we published a new strain library containing bacterial and fungal whole‐cell biocatalysts showing the ability to efficiently hydroxylate several substrates of different molecular weights and chemical properties.^[23]^ Six bacterial und six fungal strains from the library were selected. Genomic analysis revealed a high number of P450s present in their genomes with up to 174 sequences annotated as P450s in fungi and up to 50 in bacteria (Table S1). The P450 sequences can furthermore be categorized in at least 13 different superfamilies per bacterial strain and at least 21 different superfamilies per fungal strain (Table S1). Thus, on the one hand, the selected strains provide a high diversity of present P450s. On the other hand, it has already been shown that these strains are capable to convert the steroid hormone testosterone yielding different hydroxylation metabolites.^[23]^ For these reasons, we selected these strains to test for the conversion of the structurally related secosteroids VD_2_ and VD_3_.

**Figure 1:**
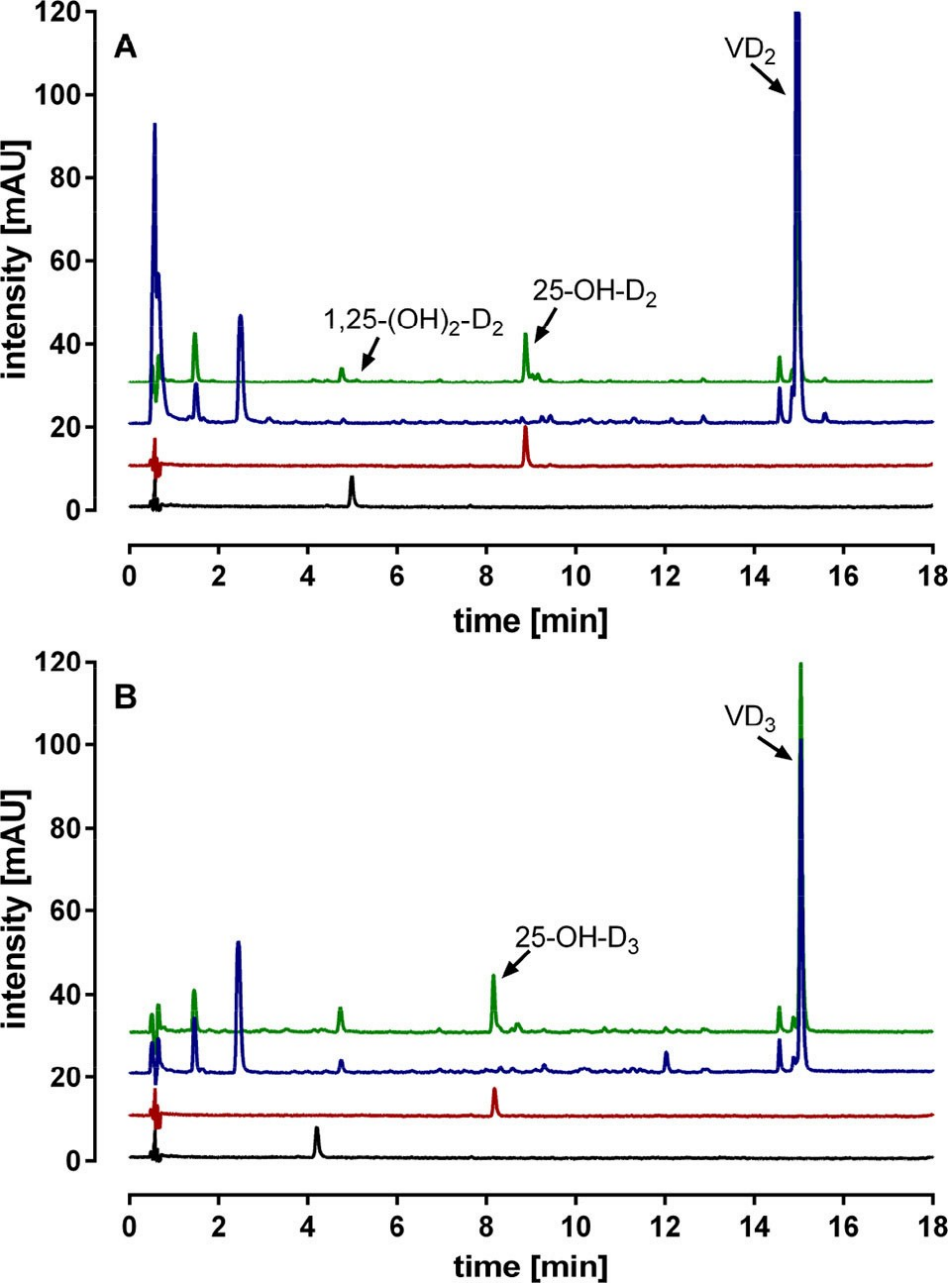
Biotransformation of VD_2_ and VD_3_ by *K. albida* in a first screening in NL148sb medium. VD_2_ or VD_3_ dissolved in DMSO were added after 48 h of cultivation to a final concentration of 0.5 mg mL^−1^. Biotransformation was performed for 48 h. A) VD_2_ conversion. B) VD_3_ conversion. The reference samples 0.00125 mg mL^−1^ 25‐OH−D_2_ or 25‐OH−D_3_ (red) and 0.00125 mg mL^−1^ 1α,25‐(OH)_2_−D_2_ or 1α,25‐(OH)_2_−D_3_ (black), biotransformation sample (green) and negative control with 0.5 mg mL^−1^ VD_2_ or VD_3_ (blue) are shown. Chromatograms are UV measurements at 265 nm.

In a first activity screening in 96‐well format, the formation of the human metabolites 25‐OH−D_2_, 25‐OH−D_3_, 1,25‐(OH)_2_−D_2_, and 1,25‐(OH)_2_−D_3_ in NL148sb medium was tested. The products were identified by comparison to reference samples of the known VD_3_ and VD_2_ analogs 25‐OH−D_3_ (*m*/*z* [*M*−H_2_O+H^+^] 383.32), 1α,25‐(OH)_2_−D_3_ (*m*/*z* [*M*−H_2_O+H^+^] 399.32), 25‐OH−D_2_ (*m*/*z* [*M*−H_2_O+H^+^] 395.32), and 1α,25‐(OH)_2_−D_2_ (*m*/*z* [*M*−H_2_O+H^+^] 411.32). An initial screening revealed *K. albida* to be able to hydroxylate both VD_2_ and VD_3_ in the C25 position (Figure 1). In addition to the single hydroxylation, tiny amounts of the double‐hydroxylation product of VD_2_, 1,25‐(OH)_2_−D_2_, were detected. The double‐hydroxylation product of VD_3_ was, however, not detected.

**Figure 2:**
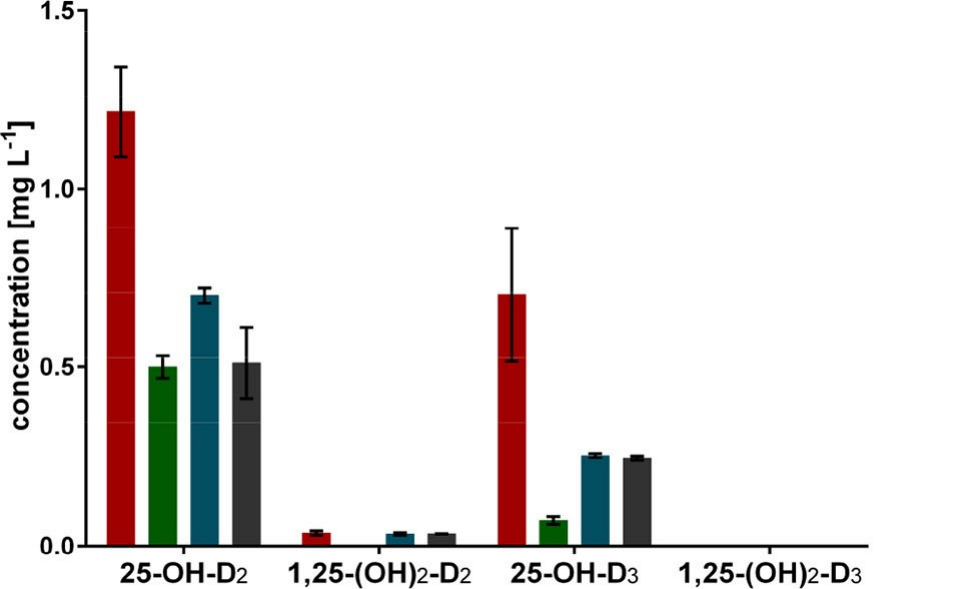
Product formation after cultivation in different media. Formation of the single‐hydroxylation products 25‐OH−D_2_ and 25‐OH−D_3_ and the double‐hydroxylation products 1,25‐(OH)_2_−D_2_ and 1,25‐(OH)_2_−D_3_ was verified by comparison to reference compounds. LB (red), TB (green), NL148sb (blue) and GYM (gray). Values are the means of duplicates.

As it has been shown before that P450 expression can depend on the used carbon source and the type of resulting metabolism,^[24]^ a medium variation was performed to enhance hydroxylation of VD_2_ and VD_3_. After cultivation in different complex media, *K. albida* produced small amounts of both single‐hydroxylation products and the double‐hydroxylation product 1,25‐(OH)_2_−D_2_, but the double‐hydroxylation product 1,25‐(OH)_2_−D_3_ was not detectable. Even though, the medium variation did not change the product range, cultivation in LB medium resulted in a two times higher concentration of 25‐OH−D_2_ (1.24 mg L^−1^) and 25‐OH−D_3_ (0.72 mg L^−1^) compared to the initial cultivation in NL148sb (Figure 2).

Overall, the biotransformation was still not efficient. It was observed that the concentration of solved VD_2_ and VD_3_ varied between the different replicates, even though it was added to the same final concentration of 0.5 mg mL^−1^. As VD_2_ and VD_3_ are nearly insoluble in water, it was assumed that most of the substrate precipitated during the cultivation and was therefore not available for the biocatalyst.

### Solubilizers improved the solubility of the substrates in medium

Steroids and the related secosteroids exhibit in general very low solubility in aqueous solution. For an efficient biotransformation, it is necessary to use concentrations above the solubility limit; however, this results in precipitation of the substrate. In the pharmaceutical, food and cosmetics industry cyclodextrins, surfactants and emulsifiers, such as polysorbates and poly(ethylene glycol) 200 (PEG200), find a broad application as formulators and are listed in the US FDA's Inactive Ingredient Database (IID). Furthermore, their applicability in biocatalysis and biotransformations was described to improve substrate solubility of highly hydrophobic compounds, such as steroids.[^17^, ^18^, ^25^]

**Figure 3:**
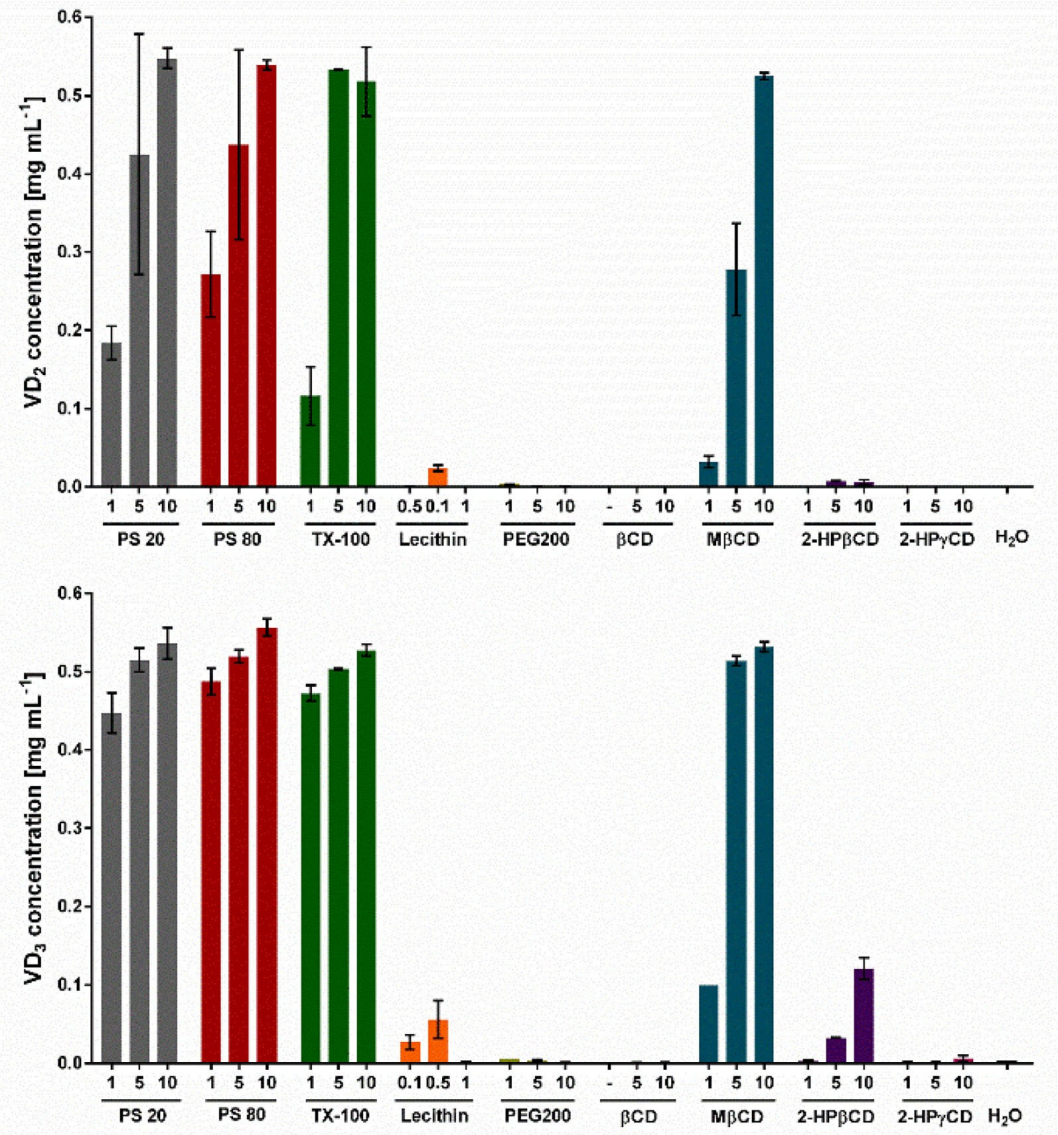
Determination of the solubility of VD_2_ and VD_3_ in medium in the presence of different solubilizers. VD_2_ or VD_3_ was added to a final concentration of 0.5 mg mL^−1^. The concentration was determined after the addition of 1, 5, and 10 % PS 20, PS 80, TX‐100, PEG200, βCD, MβCD, 2‐HPβCD, or 2‐HPγCD and 0.1, 0.5, or 1 % lecithin. As a control water was added instead of a solubilizer. Values are the means of duplicates.

In a first experiment, the solubility of VD_2_ and VD_3_ in medium in combination with different sugars, cyclic oligosaccharides, and emulsifiers as solubilizers was tested. Linear oligosaccharides and their derivatives, the sugar alcohols, have been observed to form supramolecular complexes with poorly soluble substrates similar to cyclic oligosaccharides leading to an increased solubility and stability.^[26]^ The addition of the sugars fructose and sucrose and the sugar alcohol mannitol did not enhance the solubility of VD_2_ and VD_3_ in media (data not shown). In addition, polysorbate 20 (PS 20) and polysorbate 80 (PS 80), Triton X‐100 (TX‐100), PEG200, lecithin and the cyclic oligosaccharides and derivatives β‐cyclodextrin (βCD), methyl β‐cyclodextrin (MβCD), 2‐hydroxypropyl β‐cyclodextrin (2‐HPβCD), and 2‐hydroxypropyl γ‐cyclodextrin (2‐HPγCD) were tested. Solubility was analyzed by quantifying the VD_2_ and VD_3_ concentration in medium in comparison to a sample without solubilizer but addition of H_2_O. The highest concentrations of VD_2_ and VD_3_ were obtained in medium with the PS 20, PS 80 and TX‐100 with addition of 5 and 10 % (*v*/*v*), reaching a dissolved VD_2_ and VD_3_ concentration of approximately 0.5 mg mL^−1^ (Figure 3). Furthermore, VD_2_ and VD_3_ concentrations of 0.5 mg mL^−1^ were achieved in medium supplemented with 10 % (*w*/*v*) MβCD. In medium containing lecithin, PEG200, βCD, 2‐HPβCD and 2‐HPγCD no or low concentrations of VD_2_ and VD_3_ were detected.

### Supplementation with solubilizers yielded an improved product formation in whole‐cell biotransformations

**Figure 4:**
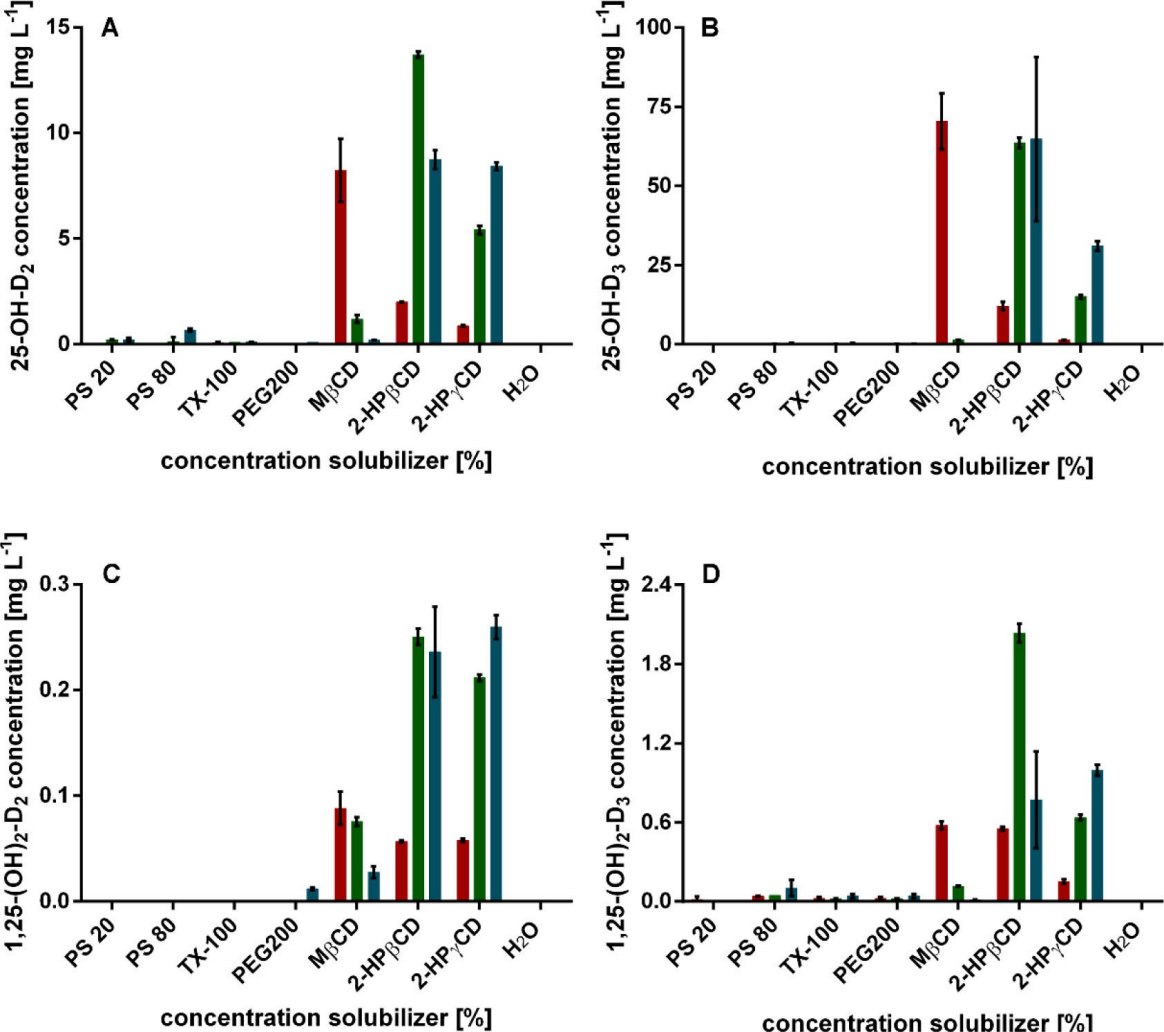
Product concentrations after biotransformation with *K. albida* in the presence of solubilizers. Product concentrations of A) 25‐OH−D_2_, B) 25‐OH−D_3_, C) 1,25‐(OH)_2_−D_2_, and D) 1,25‐(OH)_2_−D_3_ in the presence of the different solubilizers PS 20, PS 80, TX‐100, PEG200, MβCD, 2‐HPβCD, and 2‐HPγCD. As a control H_2_O was added rather than solubilizer. Concentrations of 1 (red), 5 (green), and 10 % (blue) were added. Values are the means of duplicates.

In addition to the substrate solubility in pure medium, the influence of the solubilizers on the whole‐cell biotransformation was investigated. Contrary to improved solubility of VD_2_ and VD_3_ in medium supplemented with PS 20, PS 80 and TX‐100, no product formation was observed in biotransformation samples (Figure 4). Product formation was also not detectable after the addition of PEG200. In contrast, the addition of cyclodextrins resulted in product formation, especially after the addition of MβCD, 2‐HPβCD, and 2‐HPγCD. Cyclodextrins are widely used in bioconversion and fermentation processes to enhance solubility in aqueous solution and chemical stability of hydrophobic substrates by the formation of inclusion complexes. Furthermore, many of the known cyclodextrins have a good biocompatibility with microorganisms.^[27]^ The addition of 5 % (*w*/*v*) 2‐HPβCD as a solubilizer yielded the highest product concentrations taking both single‐ and double‐hydroxylation products of VD_2_ and VD_3_ into account (Figure 4). A product concentration of 13.7 mg L^−1^ 25‐OH−D_2_ and 63.7 mg L^−1^ 25‐OH−D_3_ was reached corresponding to 3 % and 13 % yield, respectively. The doubly hydroxylated products 1,25‐(OH)_2_−D_2_ and 1,25‐(OH)_2_−D_3_ were produced with 0.25 and 2.0 mg L^−1^. The highest concentration of the singly hydroxylated product 25‐OH−D_3_ was detected after supplementation of 1 % (*w*/*v*) MβCD, with a final product concentration of 70.4 mg L^−1^ corresponding to 14 % yield. By addition of 1 % (*w*/*v*) MβCD the concentration of the doubly hydroxylated product, however, was lower with only 0.58 mg L^−1^. This is in contrast to the conversion of VD_2_ resulting in lower product concentrations for biotransformations supplemented with MβCD compared to 2‐HPβCD. Higher concentrations of MβCD (5 and 10 % (*w*/*v*)), which showed a better solubility of the substrates in medium before, resulted in a nearly total loss of activity. 2‐HPγCD, especially in high concentrations of 10 % (*w*/*v*) showed a comparable positive effect on the biotransformation as 2‐HPβCD. The addition of 10 % (*w*/*v*) 2‐HPγCD, however, yielded in overall lower concentrations compared to 2‐HPβCD. Product concentrations of 8.4 mg L^−1^ 25‐OH−D_2_, 31.0 mg L^−1^ 25‐OH−D_3_, 0.26 mg L^−1^ 1,25‐(OH)_2_−D_2_ and 1.0 mg L^−1^ 1,25‐(OH)_2_−D_3_ were reached.

In addition to the human metabolites, also the formation of other potential hydroxylation products was evaluated. As the addition 5 % (*w*/*v*) 2‐HPβCD resulted in the overall highest product concentrations, these biotransformation samples were analyzed with regard to additional hydroxylation products (Table 1). Besides the human metabolites 25‐OH−D_3_ and 25‐OH−D_2_, which made up the biggest amount of the overall formed single‐hydroxylation products (based on total peak area), three additional potential single‐hydroxylation products of VD_2_ and two of VD_3_ were formed. Further products were detected, which might be the doubly hydroxylated products of VD_2_ and VD_3_. In addition to the human metabolites 1,25‐(OH)_2_−D_2_ and 1,25‐(OH)_2_−D_3_, four potential double‐hydroxylation products of both VD_2_ and VD_3_ deduced from the *m*/*z* values were found. Based on total peak area, the concentration of these additional products was as high or even higher, as the concentration of the human metabolites.

**Table 1:** Hydroxylations of VD_2_ and VD_3_ identified after the biotransformation with *K. albida*. Samples were supplemented with 5 % (*w*/*v*) 2‐HPβCD. Areas were determined at a wavelength of 265 nm.

Substrate	*m*/*z*^[a]^	Suggested type of reaction	t_R_ [min]	Area [mAU*min]
VD_2_	395.32	single hydroxylation 25‐OH−D_2_^[b]^	9.1	135.2
	395.32	single hydroxylation	9.4	6.7
	395.32	single hydroxylation	9.5	7.0
	395.32	single hydroxylation	9.9	4.9
	411.32	double hydroxylation	4.3	11.4
	411.32	double hydroxylation	5.2	3.0
	411.32	double hydroxylation 1,25‐(OH)_2_−D_2_^[b]^	5.3	3.0
	411.32	double hydroxylation	5.5	1.6
	411.32	double hydroxylation	5.8	1.7
VD_3_	383.32	single hydroxylation 25‐OH−D_3_^[b]^	8.4	760.5
	383.32	single hydroxylation	8.8	105.5
	383.32	single hydroxylation	9.2	26.9
	399.32	double hydroxylation	3.8	44.0
	399.32	double hydroxylation	4.3	32.2
	399.32	double hydroxylation	4.4	30.3
	399.32	double hydroxylation 1,25‐(OH)_2_−D_3_^[b]^	4.6	4.5
	399.32	double hydroxylation	5.1	11.6

[a] [*M*−H_2_O+H^+^] of metabolite. [b] product was verified by a chemical standard.

## Discussion

In a previous genome mining linked to an activity screening, we were able to identify a number of previously unknown biocatalysts for oxyfunctionalization reactions.^[23]^ From this strain library, biocatalysts with a broad product range and high conversion of the steroid hormone testosterone were selected and tested for the conversion of VD_2_ and VD_3_. From twelve tested strains (six fungal and six bacterial), one strain, the ascomycete *K. albida*, was identified to catalyze VD_2_‐and VD_3_‐specific hydroxylations. The human single‐hydroxylation products 25‐OH−D_3_ and 25‐OH−D_2_ and small amounts of the double‐hydroxylation product 1,25‐(OH)_2_−D_2_ were identified in a first screening. *K. albida* is one of eight members of the genus *Kutzneria*, which is a minor branch of the *Pseudonocardiaceae* family^[28]^ and is therefore related to *Pseudonocardia autotrophica* from the genus *Pseudonocardia* that is known to catalyze hydroxylations of VD_3_. Genome sequencing of *K. albida* revealed that 14 % of the chromosome is composed of secondary metabolism gene clusters,^[29]^ which complies with a large number of P450s present in this strain (Table S1). Previous screening of *K. albida* showed a broad hydroxylation potential in the conversion of seven tested pharmaceutical compounds among other complex molecules, such as cyclosporine A, tamoxifen, and ritonavir.^[30]^ Categorization of the 50 encoded P450s in superfamilies revealed the CYP107 family, the family of the known VD_3_ hydroxylase Vdh from *P. autotrophica*, as most abundant (Table S2).^[31]^

The first screening yielded low concentrations of the single‐hydroxylation products and the double‐hydroxylation product 1,25‐(OH)_2_−D_2_. The double‐hydroxylation product 1,25‐(OH)_2_−D_3_ was not detected. Changing the cultivation medium to LB medium did not result in additional double‐hydroxylation products, however yielded a doubled amount of the single‐hydroxylation products. It was generally assumed that the availability of the substrate to the biocatalyst was limited due to the low solubility of VD_2_ and VD_3_. To improve the substrate solubility and therefore availability to the biocatalyst, the application of different solubilizers was tested to finally improve productivity.

Medium supplemented with PS 20, PS 80 and TX‐100 resulted in a drastically increased solubility of VD_2_ and VD_3_. The addition of MβCD (5 and 10 % (*w*/*v*)) also enhanced the concentration of VD_2_ and VD_3_ in medium. In comparison, supplementation with 2‐HPβCD and 2‐HPγCD and low concentration of MβCD (1 % (*w*/*v*)) resulted in a small increase of VD_2_ and VD_3_ solubility. In contrast, lecithin, PEG200 and β‐CD did not enhance the solubility at all. In general, the introduction of hydroxypropyl or methyl groups to the hydroxyl groups of cyclodextrins is known to enhance water solubility compared to the unsubstituted natural cyclodextrins.^[32]^ This was consistent with the obtained results.

Although the substrates were well soluble in medium supplemented with PS 20, PS 80 and TX‐100, the substrates were not converted in reaction solutions containing these solubilizers. 2‐HPβCD and 2‐HPγCD and low concentrations of MβCD, however, were shown to have a positive effect on the biocatalytic activity of *K. albida*. High concentrations of MβCD resulted in a total loss of the biocatalytic activity, just as the previously mentioned solubilizers. These results suggest that either high concentrations of dissolved VD_2_ and VD_3_ or the solubilizers themselves have an inhibitory or toxic effect on the biocatalyst *K. albida* and therefore do not promote a better substrate conversion in the biotransformation. Inhibitory or toxic effects of the substrate or product on the biocatalyst as limiting factor for a biotransformation have been analyzed in literature before.^[30]^ To overcome such limitations, the use of cyclodextrins has been discussed.^[33]^ Besides the already mentioned enhancement of solubility, cyclodextrins also provide a reduction of substrate toxicity by forming a complex with the substrate molecule. Results for MβCD, however, contradict this theory. Even though all used concentrations of MβCD enhance substrate solubility, high concentrations result in a total loss of product formation. In contrast, biotransformations with low MβCD concentrations led to increased product formation. As MβCD is known to form complexes with hydrophobic molecules, thus lowering the substrate toxicity even at high dissolved concentrations, the results indicate that the reduced product formation in the biotransformations is rather a result of toxic effects of the solubilizers themself.

This is also supported by the results of the other solubilizers. Polysorbates and TX‐100 possess a non‐ionic head unit and in general belong to the “mild” surfactants. They are used for the solubilization of proteins by forming detergent‐protein complexes without loss of the biological activity.^[34]^ On cell membranes, however, large amounts of these detergents were described to have disrupting properties.^[35]^ Possibly, the used concentrations of the detergents were toxic to the cells. Concerning cyclodextrins, several studies have described an inhibitory effect on the catalytic activity of whole‐cell biocatalysts. Zehentgruber et al. observed an inhibitory or toxic effect of the three different cyclodextrins α‐, γ‐ and 2‐HPγCD during the biotransformation of progesterone with recombinant *Schizosaccharomyces pombe*.^[17]^ In another study, β‐cyclodextrin was described to be more toxic for *Escherichia coli* cells than γ‐cyclodextrin and comparing the chemically substituted cyclodextrin derivatives, MβCD was more toxic to *E. coli* than 2‐HPγCD.^[36]^ It was generally suggested that especially alkylated CD derivatives, such as MβCD, can form complexes with biomolecules in the membrane and therefore enhance membrane permeability.^[37]^ Due to this property, MβCD is also used to deplete cholesterol from lipid rafts.^[38]^ Cell‐wall permeability, however, was also observed in the presence of 2‐HPβCD.^[39]^ Comparing the two actinobacteria *Arthrobacter simplex* and *Mycobacterium* sp. NRRL B‐3683, it was observed that the impact of 2‐HPγCD on growth, biocatalytic activity, and cell integrity significantly differs and is dependent on the composition of the cell wall and cell membrane. Concerning *K. albida*, high concentrations of 2‐HPβCD and 2‐HPγCD seem to be better tolerated in comparison to MβCD.

Even though all cyclodextrins could improve product formation in the biotransformation, clear differences between the cyclodextrins could be observed, with 5 % (*w*/*v*) 2‐HPβCD resulting in the highest overall product concentration considering both single‐ and double‐hydroxylation products. In general, product formation for 2‐HPβCD was higher than the formation for respective concentrations of 2‐HPγCD. This may be related to the different cavity sizes of 2‐HPβCD and 2‐HPγCD, which is determined by the number of α‐1‐4‐linked glucose units (αCD=6, βCD=7, γCD=8).^[40]^ The cavity size is known to be most important for interaction with the substrate. β‐Cyclodextrins are the most commonly used cyclodextrins with generally higher complexing abilities.^[20]^ Complexation of VD_3_ with 2‐HPβCD was described to result in a 43.5‐times increase of the solubility (from 0.23 to 10 mg mL^‐1^).^[41]^ Several studies compared the complexation capacities of 2‐HPβCD and 2‐HPγCD with different steroids. Investigation of the complexation of the steroid danazol for example resulted in a higher solubility and stability constant (*K*_1:1_) in complex with 2‐HPβCD compared to 2‐HPγCD. A stronger complex formation with βCD was also observed for the steroid prednisolone. The bulkier 6α‐methyl prednisolone, however, favored complexation with γCD.^[42]^ Thus, a stronger complex formation of VD_2_ or VD_3_ and 2‐HPβCD compared to 2‐HPγCD might have resulted in higher available substrate concentrations and consequently higher product yields. Additionally, the binding geometry of the substrate within the cavity might have an influence on the accessibility of the substrate to the biocatalyst. To determine the binding affinities between VD_2_ or VD_3_ and 2‐HPβCD, detailed phase‐solubility studies and complex structure analyses should be performed.

The positive effect of cyclodextrins on the biotransformation might to some extend be a result of cell wall destabilization facilitating the mass transfer of the substrate and product through the membrane and cell wall. To further improve the biotransformation and to overcome possible mass transfer limitations a combination of membrane permeabilizing substances and cyclodextrins could be tested. Polysorbates and TX‐100, known to exhibit permeabilizing activity, however showed a negative effect on cell viability. Organic solvents or chelating agents, such as hexadodecyl trimethyl ammonium bromide^[17]^ or EDTA^[43]^ can be used to weaken the membrane. In addition, the antimicrobial peptide nisin was reported to significantly improve 25‐OH−D_3_ by recombinant *Rhodococcus erythropolis* presumably due to pore formation.^[44]^ For gram‐negative whole‐cell biocatalysts, polymyxin B was shown to increase biotransformation rates of low soluble substrates by membrane permeabilization.[^43^, ^45^]

Regardless of possible influences of substrate solubility, availability and solubilizer toxicity, *K. albida* proved to be a potential novel biocatalyst for the one step production of the human metabolites 25‐OH−D and 1,25‐(OH)_2_−D. Especially, the metabolite 25‐OH−D_3_ was produced with a 14 % yield and a productivity of 1.47 mg L^−1^ h^−1^. The productivity reached by *K. albida* is within the same order of magnitude as other biotransformations with actinomycetes.^[46]^ Besides the human metabolites, *K. albida* was able to form several other potential hydroxylation products. The hydroxylation in the C25 position is the main single‐hydroxylation product for both VD_2_ and VD_3_ and only smaller concentrations of other potential single‐hydroxylation products were formed. Especially, several other potential double‐hydroxylation products with higher intensities based on the HPLC peak area than the human metabolites 1,25‐(OH)_2_−D were formed. These analogs can offer an alternative to the human metabolites with altered application properties. More than 3000 synthetic VD_3_ analogs have been developed to access metabolites with advanced properties for the therapeutic application in hyperproliferative diseases, cancer or osteoporosis.[^5^, ^47^] 20−OH−D_3_ for example has been developed to display antiproliferative activity, antileukemic and tumorostatic effects.^[48]^ At the same time, this analog showed less toxic side effects, such as hypercalcemia, compared to the human equivalents. The one‐step production of VD_2_ and VD_3_ derivatives other than the human metabolites by microbial biotransformation would offer great advances compared to complex chemical syntheses. For this, purification and characterization of the unknown metabolites is needed. To exploit the full biocatalytic potential and to further improve substrate conversion, optimization of VD_2_ and VD_3_ addition time and loading will be needed. Furthermore, to improve the productivity, an optimal bioconversion time has to be determined. In a next step, therefore a scale‐up of the reaction to a stirred system should be investigated to develop a preparative production of hydroxylated VD_2_ and VD_3_ derivatives with *K. albida*.

In addition, the identification of responsible P450s in the genome of *K. albida* and recombinant expression would contribute to further increase productivities. This however might become challenging since *K. albida* is not an industrial host and the underlying pathways are not well characterized. Yet, the analysis of the genomic information for present P450s and categorization into superfamilies as provided in Table S2 will guide the identification of the genes coding for vitamin D hydroxylating enzymes.

## Conclusion

In this study, we have identified a novel actinomycete *K. albida* that is able to introduce single and double hydroxylations into the substrates VD_2_ and VD_3_. *K. albida* was thereby able to form the human metabolites 25‐OH−D_2_, 25‐OH−D_3_, 1,25‐(OH)_2_−D_2_, and 1,25‐(OH)_2_−D_3_. The addition of cyclodextrins to the medium significantly improved the product yield with up to 100‐fold higher concentrations of 25‐OH−D_3_ and tenfold higher concentrations of 25‐OH−D_2_. Highest product concentrations of the 25‐OH−D_3_ metabolite were reached after supplementation of the medium with 1 % (*w*/*v*) MβCD. A product concentration of 70.4 mg L^−1^, corresponding to a yield of 14 %, and a productivity of 1.47 mg L^−1^ h^−1^ were reached. The highest concentrations of both single‐ and double‐hydroxylation products, of VD_2_ and VD_3_ were achieved with the addition of 5 % (*w*/*v*) 2‐HPβCD. Concentrations of 13.7 mg L^−1^ 25‐OH−D_2_ and 63.7 mg L^−1^ 25‐OH−D_3_ were formed corresponding to yields of 3 and 13 %, respectively. The double‐hydroxylation products 1,25‐(OH)_2_−D_3_ and 1,25‐(OH)_2_−D_2_ were formed with 2.0 and 0.25 mg L^−1^, respectively. Besides the human metabolites, *K. albida* was also able to form other potential single‐ and double‐hydroxylation products. In particular, several additional potential double‐hydroxylation products were identified that could be structurally analyzed in a next step and tested with regard to new therapeutic agents for alternative or improved biological activities. The efficient hydroxylation capabilities elucidate the potential of *K. albida* as a novel biocatalyst for the microbial production of VD_2_ and VD_3_ metabolites.

## Experimental Section

Screening for strains catalyzing the hydroxylation VD_2_ and VD_3_: 12 stains (Table 2) were tested for the biotransformation of VD_2_ and VD_3_. Growth of bacterial strains was performed in standard NL148 medium for pre cultivation and NL148sb medium for the main culture. For cell growth of the fungi NL148s medium was used. Precultures of microbial strains were grown in 100 mL shake flasks (20 % filling volume) for 3 days, 24–28 °C, and 180 rpm (2.5 cm shaking diameter). Main cultures were grown in 500 mL shake flasks (12 % filling volume) for 3 days, 24–28 °C, and 180 rpm (2.5 cm shaking diameter). The biotransformations were performed in 96‐Deep well plates of the System Duetz® (Enzyscreen, Heemstede) with 500 μL filling volume, 28 °C, 200 rpm (5 cm shaking diameter) for 48 h. 495 μL of the culture was transferred to 96−Deep well plates and 5 μL of substrate was added to a final substrate concentration of 0.5 mg mL^−1^. After 48 h of biotransformation, cultures were harvested and extracted three times with an equal amount *n*‐hexane. Samples were evaporated to dryness and resolved in 500 μL methanol for LC‐MS analysis.

**Table 2:** Strains tested for VD_2_ and VD_3_ conversion.

	Strain number	Name
Fungal strains	CBS‐126508	*Colletotrichum fioriniae*
	CBS‐130836	*Colletotrichum graminicola*
	CBS‐131301	*Colletotrichum sublineola*
	CBS‐208.87	*Eutypa lata*
	DSM‐898	*Penicillium oxalicum*
	UAMH 1704	*Uncinocarpus reesii*
Bacterial strains	DSM‐43870	*Kutzneria albida*
	DSM‐43936	*Actinomadura rifamycini*
	DSM‐43827	*Actinosynnema mirum*
	DSM‐44213	*Amycolatopsis japonica*
	DSM‐44437	*Lentzea albida*
	DSM‐45390	*Saccharomonospora marina*

Media variation to optimize biotransformation: Cultivation of *K. albida* was performed in MTP‐48‐well‐FlowerPlates in the BioLector (m2p‐labs, Baesweiler). Plates were filled with 787 μL medium and inoculated with 13 μL of a preculture grown in 20 mL NL148 medium at 28 °C for three days. Cultivation in the BioLector was performed with a shaking frequency of 1500 min^−1^ at 28 °C for 112 h. Biomass concentrations were quantified by scattered light intensity (with a gain of 20). Substrates (VD_2_ and VD_3_) were added 30 h after inoculation to a final concentration of 0.5 mg mL^−1^. Tested media were glucose‐yeast‐malt extract (GYM), terrific broth (TB), NL148sb and lysogeny broth (LB). Recipes of used media are listed in the Supporting Information. Samples were extracted three times with an equal amount *n*‐hexane. Samples were evaporated to dryness and resolved in 408 μL methanol for LC–MS analysis.

Testing substrate solubility in the presence of solubilizers: Solubility of VD_2_ and VD_3_ in LB was tested with various solubilizers in MTP‐48‐well‐FlowerPlates in the BioLector. LB was supplemented with 1 % (*v*/*v*), 5 % (*v*/*v*), 10 % (*v*/*v*) PS 20, PS 80, PEG200, and TX‐100 (from a 100 % stock solution) and 1 % (*w*/*v*), 5 % (*w*/*v*), 10 % (*w*/*v*) βCD, MβCD, 2‐HPβCD, and 2‐HPγCD (from a 555 g L^−1^ stock solution). VD_2_ and VD_3_ were added to a final concentration of 0.5 mg mL^−1^. The solutions were incubated with a shaking frequency of 1,200 min^−1^ at 28 °C for 48 h. Samples were centrifuged at 3,355 g for 5 min and the supernatant was transferred to a vial. An equal amount of 50 % (*v*/*v*) acetonitrile was added prior to LC‐MS measurement.

Testing compatibility of solubilizers with the biotransformation with *K. albida*: Cultivation of *K. albida* was performed in MTP‐48‐well‐FlowerPlates in the BioLector. Plates were filled with 627 μL LB medium and inoculated with 13 μL of a preculture grown in 20 mL LB medium at 28 °C for three days. Cultivation was performed with a shaking frequency of 1200 min^−1^ at 28 °C. Scattered Light was measured with a gain of 20. After 48 h of cultivation 1 (*v*/*v*), 5 (*v*/*v*), or 10 % (*v*/*v*) PS 20, PS 80, PEG200, and TX‐100 (from a 100 % stock solution) and 1, 5, or 10 % (*w*/*v*) βCD, MβCD, 2‐HPβCD, and 2‐HPγCD (from a 555 g L^−1^ stock solution) was added. The control samples without solubilizer were filled up to 800 μL with water. VD_2_ and VD_3_ were added to a final concentration of 0.5 mg mL^−1^. Cultivation was performed for 48 h with a shaking frequency of 1,200 min^−1^ at 28 °C. After 48 h of cultivation samples were transferred in 1.5 mL tubes and centrifuged at 9,615 g and room temperature for 10 min. The supernatant was transferred to a vial and diluted 1 : 2 with 50 % (*v*/*v*) acetonitrile. Samples were filtered with a 0.45 μm pore size polyamide filter prior to LC–MS measurement.

Analytics of substrate solubility and biotransformation samples: Analysis of the samples was performed with an 1260 Infinity LC system (Agilent) combined with a compact quadrupole time of flight (Q‐TOF) mass spectrometer (Bruker Daltonics) using a Nucleoshell RP18 column, 2.0×100 mm, 2.7 μm (Macherey‐Nagel). The flow rate was 0.4 mL min^−1^. The column temperature was set to 40 °C. The following gradient of 0.1 % formic acid (*v*/*v*, solvent A) and 100 % acetonitrile (solvent B) was used: 0–12 min: 50 to 100 % B, 12–15 min: 100 % B. The Q‐TOF was interfaced with electron spray ionization (ESI). Following ESI parameters were set: drying gas temperature: 220 °C, nebulizer pressure: 4.8 bar, drying gas flow: 12 L min^−1^, capillary voltage: 4,500 V, end plate offset: 500 V. Analytes were detected in a range of *m*/*z* 100 to 800. UV detection was performed at a wavelength of 265 nm. For the verification of the main human metabolites analytical standards of 25‐OH−D_2_, 25‐OH−D_3_, 1α,25‐(OH)_2_−D_2_, 1α,25‐(OH)_2_−D_3_ obtained from Sigma‐Aldrich in analytical grade were measured.

## Conflict of interest

The authors declare no conflict of interest.

## Supporting information

As a service to our authors and readers, this journal provides supporting information supplied by the authors. Such materials are peer reviewed and may be re‐organized for online delivery, but are not copy‐edited or typeset. Technical support issues arising from supporting information (other than missing files) should be addressed to the authors.

Supplementary
