# Supplementary material for: Investigation of Vitamin D2 and Vitamin D3 Hydroxylation by Kutzneria albida
Source: Chembiochem. 2021 May 4;22(13):2266–74. doi: 10.1002/cbic.202100027 (PMC8359954; doi:10.1002/cbic.202100027)
Supplement: Supplementary file 1 — Supplementary [file CBIC-22-2266-s001.pdf]

ChemBioChem

Supporting Information

Investigation of Vitamin D_2 and Vitamin D_3 Hydroxylation by *Kutzneria albida*

Lisa Marie Schmitz, Alina Kinner, Kirsten Althoff, Katrin Rosenthal, and Stephan Lütz*

Supplementary Materials

Glucose-yeast-malt extract medium (GYM): 4.0 g L^{-1} glucose, 4.0 g L^{-1} yeast extract, 10.0 g L^{-1} malt
extract, pH 7.2

Terrific broth medium (TB): 12 g L^{-1} tryptone, 24 g L^{-1} yeast extract, 5 g L^{-1} glycerol, 2.31 g L^{-1} potassium dihydrogen phosphate, and 12.54 g L^{-1} dipotassium hydrogen phosphate

NL148 medium: 22 g L^{-1} glucose, 8 g L^{-1} casein peptone, 4 g L^{-1} meat extract, 0.5 g L^{-1} yeast extract, 1.5 g L^{-1} sodium chloride, 21 g L^{-1} 3-(*N*-morpholino)propanesulfonic acid (MOPS), 0.1 mg L^{-1} boric acid, 5 mg L^{-1} iron sulphate, 0.05 mg L^{-1} potassium iodide, 2 mg L^{-1} cobalt chloride × 6 H_2O, 0.2 mg L^{-1} copper sulfate × 5 H_2O, 2 mg L^{-1} manganese chloride × 4 H_2O, 4 mg L^{-1} zinc sulfate × 7 H_2O, and 1 µL sulfuric acid (97 %), pH 6.5

NL148sb medium: 20 g L^{-1} soluble starch, 4 g L^{-1} casein peptone, 4 g L^{-1} soya peptone, 4 g L^{-1} meat extract, 0.5 g L^{-1} yeast extract, 1.5 g L^{-1} sodium chloride, 21 g L^{-1} MOPS, 0.1 mg L^{-1} boric acid, 5 mg L^{-1} iron sulphate, 0.05 mg L^{-1} potassium iodide, 2 mg L^{-1} cobalt chloride × 6 H_2O, 0.2 mg L^{-1} copper sulfate × 5 H_2O, 2 mg L^{-1} manganese chloride × 4 H_2O, 4 mg L^{-1} zinc sulfate × 7 H_2O, and 1 µL sulfuric acid (97 %), pH 7.5

Lysogeny broth medium (LB): 10 g L^{-1} tryptone, 5 g L^{-1} yeast extract, 10 g L^{-1} sodium chloride, pH 7.0

Supplementary Results

Table S1: Genomic analysis of the tested bacteria and fungi.

Name of microorganism	NCBI Taxonomy ID	number of gene sequences annotated to P450s (NCBI/uniprot)	number of superfamilies (>40%)[1] (based on CYPED)	number of homologous families (> 55%)[1] (based on CYPED)
Eutypa lata UCR-EL1	1287681	174	31	21
Colletotrichum fioriniae PJ7	1445577	130	81	33
Colletotrichum graminicola M1.001	645133	124	62	28
Colletotrichum sublineola	1173701	118	62	30

Kutzneria albida DSM 43870	1449976	50	13	7
Penicillium oxalicum 114-2	933388	42	26	18
Actinomadura rifamycini DSM 43936	31962	40	21	16
Lentzea albida DSM 44437	65499	38	15	9
Actinosynnema mirum DSM 43827	40567	37	17	6
Amycolatopsis japonica DSM 44213	208439	37	24	13
Saccharomonospora marina XMU15	632569	28	20	14
Uncinocarpus reesii UAMH 1704	336963	32	21	12

Table S2. Genomic analysis of *K. albida.* The P450s were categorized in superfamilies according to D.R. Nelson.[2] Sequence identity to Vdh was analyzed by protein BLAST.

Strain (genome accession number)	distribution of present P450 families	gene names as locus_tags	Length	sequence identity [%] to Vdh (GenBank accession: OSY34502.1)
Kutzneria albida	32% CYP107	KALB_104	394	36.06
DSM 43870		KALB_664	416	38.93
(NZ_CP007155.1)		KALB_1349	378	43.63
		KALB_1434	393	38.78
		KALB_1618	401	43.27
		KALB_1619	362	42.74
		KALB_3912	469	41.86
		KALB_3944	398	37.18
		KALB_3945	426	47.50
		KALB_4121	408	42.02
		KALB_4776	421	34.29
		KALB_6464	386	41.75
		KALB_7081	403	44.03
		KALB_7250	397	33.97
		KALB_7453	395	40.31
		KALB_7726	399	42.11
	18% CYP105	KALB_1475	400	39.27
		KALB_2269	403	39.38
		KALB_3845	404	39.09
		KALB_5118	375	39.31
		KALB_5482	320	43.91
		KALB_6537	390	44.53
		KALB_6568	403	36.80
		KALB_8066	399	44.30
		KALB_8776	408	42.78

6% CYP125	KALB_2127	376	32.20
	KALB_3393	405	35.80
	KALB_3752	433	32.07
6% CYP163	KALB_5803	97	37.08
	KALB_5903	407	29.47
	KALB_6129	415	27.47
4% CYP183	KALB_1468	459	28.65
	KALB_2486	449	27.36
4% CYP102	KALB_657	1071	22.77
	KALB_3295	468	28.50
4% CYP245	KALB_3411	414	37.07
	KALB_5825	410	34.33
2% CYP113	KALB_1659	401	36.99
2% CYP116	KALB_7240	378	33.43
2% CYP140	KALB_3089	425	39.76
2% CYP154	KALB_3732	411	40.47
2% CYP164	KALB_5800	404	38.17
2% CYP165	KALB_1908	425	34.82
2% CYP244	KALB_3412	405	30.97
12% no classification possible	KALB_4365	346	28.84
	KALB_5244	389	n.d.
	KALB_5489	423	28.34
	KALB_5792	387	32.82
	KALB_6260	427	28.34
	KALB_6261	448	27.78

Growth curves for cultivation on different media from BioLector experiments

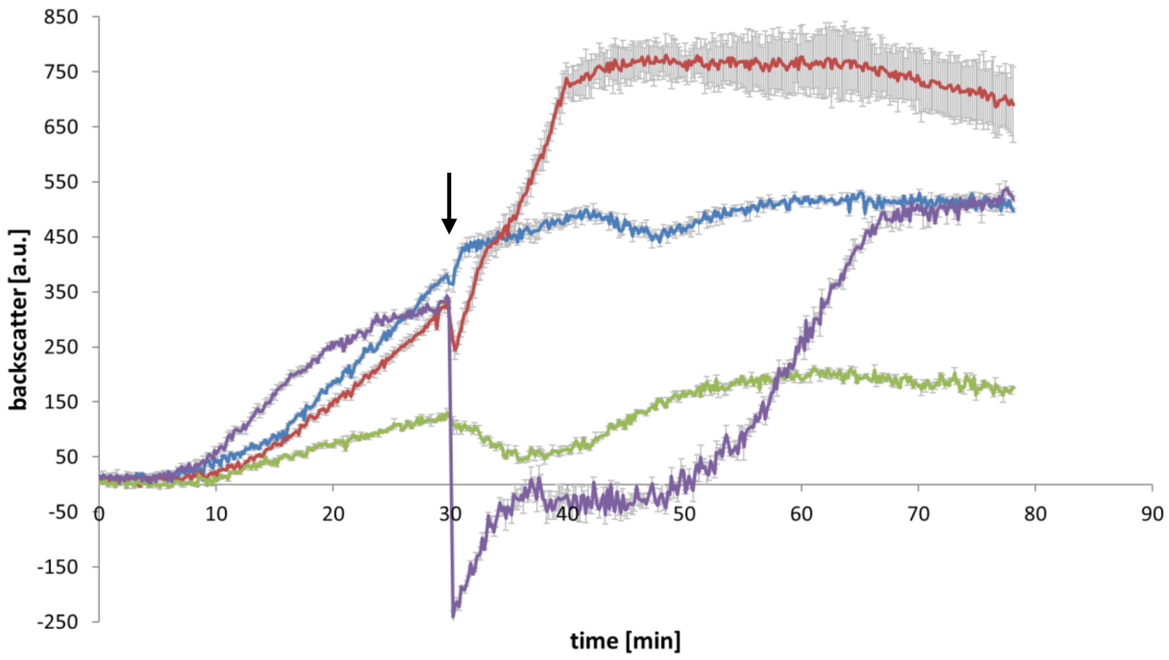

Figure S1: Growth of *K. albida* DSM 43870 on different media. Blue: LB medium, red: TB medium, green: NL148sb, purple: GYM medium. Substrate addition is marked by an arrow. Values are the mean of duplicates.

References

[1] L. M. Schmitz, J. Schäper, K. Rosenthal, S. Lütz, *ChemCatChem* **2019**, *11*, 5766-5777.

[2] D. R. Nelson, *Methods Mol. Biol.* **2006**, *320*, 1-10.
